# Spatial distribution of Q fever in sheep and goats of selective villages of Punjab Province, Pakistan

**DOI:** 10.1186/s12917-024-04421-0

**Published:** 2024-12-23

**Authors:** Freeha Amin, Shahzad Ali, Ahmad Hassan, Imran Rashid, Heinrich Neubauer, Katja Mertens-Scholz

**Affiliations:** 1 Department of Wildlife & Ecology, University of Veterinary and Animal Sciences, Lahore, Ravi Campus, Pattoki, 55300 Pakistan; 2 Department of Parasitology, University of Veterinary and Animal Sciences (UVAS), Lahore, Pakistan; 3 Friedrich-Loeffler-Institut, Institute of Bacterial Infections and Zoonoses, 07743 Jena, Germany

**Keywords:** C. burnetii, ELISA, Serology, Spatial distribution

## Abstract

This study aimed to assess the geographical distribution of Q fever in sheep and goats in different areas of Punjab, Pakistan. Three hundred blood samples of small ruminants including sheep and goats were collected from 60 villages of three districts (Okara, Kasur, and Pakpattan) of Punjab Province Pakistan and tested for the detection of anti-*Coxiella burnetii* antibodies using a commercial Indirect ELISA kit. Data related to sampling location, host species, gender, age, and GPS coordinates were collected for spatial analysis. A surface plot was created using inverse distance weight (IDW) by interpolation of the Aeronautical Reconnaissance Coverage Geographic Information system (Arc GIS). The district Kasur (14%) and the tehsil Chunian (24%) had the most prominent Q fever prevalence in both species. No ovine males were seropositive but 19.2% of male goats were seropositive. No samples of sheep younger than 1 year were found seropositive. Gender in sheep and age in goats have to be considered as significant risk factors based on multiple logistic regression analysis. Based on spatial analysis, seropositivity for *C. burnetii* antibodies was more likely observed in villages of tehsil Kasur, Pattoki, Okara, Depalpur, and Renala Khurd in the case of goats while in case of sheep villages of tehsil Chunian, Renala Khurd and Pakpattan. The main outcome of this study is that Q fever-specific antibodies of *C. burnetii* are prevalent in the goat and sheep populations of the study area and we have identified potential risk zones. The findings of this study can be used for the control of Q fever in small ruminants of the study area to minimize the risk of this zoonosis in other animals and the associated human population.

## Introduction

Query fever is caused by the worldwide distributed bacterium *Coxiella burnetii* which is a strict intracellular immotile pathogen Gram-negative gamma-proteobacterium. The pleomorphic rod-shaped *C. burnetii* is 0.2–0.4 μm in diameter and 0.4–1 μm in length. This bacterium is present in several climatic zones. It replicates within the phagolysosome of the mononuclear phagocyte but can infect various types of cells [1, 2]. *C. burnetii* can cause infection in wild and domestic animals and humans via inhaling contaminated droplets or dust particles [3]. A variety of captive animals and wildlife species including birds and mammals are also reservoirs of this pathogen [4]. Domesticated ruminants are considered the main source of human infections, but wildlife may also be involved e.g. wild rabbits [2, 5].

Ruminants can shed the agent during calving or lambing as the placenta and their birth products contain bacteria in exorbitant numbers and contamination of the surrounding environment is massive [6–8]. Spore-like forms can persist in the environment for a long time e.g. in dust particles for up to 150 days and may be transported through air from one farm to another [9, 10]. Q fever in animals does not present with specific signs and symptoms but it can cause abortion, loss of productivity and stillbirth with a great socioeconomic loss for the livestock farmers [11]. Other disorders like infertility, weak and underweight newborns, retained placenta and mastitis may also be seen. In sheep and goats, sporadic third-trimester abortions are commonly recorded cases [12]. Especially in goats Q fever can cause unbearable losses to local farmers [13]. Even though Q fever is worldwide distributed, outbreaks in humans have often been reported in European, western Asian and African countries of the temperate climate zone. Goats from local farms are considered the main source of human disease in a Dutch outbreak involving 4,000 patients [14]. Rare cases of Q fever were reported from tropical zones [15–17]. In recent years, studies from French Guiana (38.5%) and Brazil (21.4–22%) have reported high positivity rates [18–20].

Previous reports described the presence of Q fever disease in domestic and wildlife species of Pakistan [21–23]. The seroprevalence in dairy animals in Punjab, Pakistan was recorded to be 6.1% [12]. Hence, the availability of epidemiological data is scant which hampers the development of control measurements. Thus, a geographical information system that provides not only data on the spatial distribution and trends of diseases but also provides additional data on demographic, epidemiological, and environmental factors related to the occurrence and outbreaks is beneficial. This study was designed to estimate the spatial distribution of *C. burnetii* antibodies in small ruminants at the village level in specific districts of the Punjab province of Pakistan.

## Materials and methods

### Sampling area

The sampling was done in three districts of the Pakistani Punjab, i.e. Kasur (31.0896° North, 74.1240° East), Okara (30.8138°North, 73.4534°East), and Pakpattan (30.2527° North, 73.1822° East) including GIS mapping. Pakpattan is situated on the northern side of the district Okara. Agriculture is a dominant occupation of peoples which support the economic needs of 50% of the population due to the fertile soil of Pakpattan, while livestock production is a source of income for 10% population. Apart from these people are also engaged in textile, oil, rice, flour, and poultry feed mills. Kasur district is situated near the border of Ganda Singh. Due to extremely rich soils, 61% of the inhabitants make their income through animals. Sheep, goats, cattle and buffaloes are common livestock [24]. In the east of Kasur, the Okara district is located. The climate is warm (between 3^0^ and 45^0^ C) and dry. This city is renowned for dairy farms, cotton mills and agriculture [25]. The climate is variable and grasses, shrubs and scrubby vegetation take turns [26, 27]. These districts were the preferred study region due to the high density of ruminants and their intensive interaction with the human population. The geographic and climatic conditions easily permit the spread of these aerosol-borne pathogens [28]. These districts have different flora and fauna. The Beetle and Teddy goats and Kajli sheep are important breeds of sheep and goats.

### Study design and sampling strategies

A cross-sectional study was conducted to gather samples across three districts in Punjab: Kasur, Okara, and Pakpattan. Within these districts, nine tehsils Kasur, Chunian, Kot Radha Kishan, Pattoki, Okara, Depalpur, Renala Khurd, Arifwala, and Pakpattan—were included in the study. The focus was on village-based small ruminants, specifically sheep and goats. The required sample size was calculated using Thrusfield’s [29] formula, assuming a 5% margin of error, a 95% confidence level, and an estimated prevalence of 50%. This resulted in a target of 300 small ruminants. A simple random sampling approach was employed to select both villages and animals, leading to a total of sixty villages chosen using a random number table.

### GIS-based survey

Geographical coordinates of sampling sites of 60 villages were recorded. For this purpose, GIS-based surveys were conducted from 1/7/2019 to 1/7/2020 through a GPS receiver application installed in mobile phones (version 4.4.25). The geographical coordinates of the survey sites were used to construct a map to explore the spatial trends of Q fever seroprevalence in the study area. All geographical coordinates were processed through the ArcGIS 10.5.1 software.

### Data collection

Primary data of the demographic information of sheep and goats, such as geographical locations (district, tehsil and village), age (adult vs. young), gender, animal and species, were collected for each sample [30, 31]. The demographic data of animals were collected through questionnaires.

### Collection and analysis of samples

A total 300 blood samples were collected from 142 Kalji sheep and 158 goats, including 119 Beetal and 39 Teddy breeds. Among these, 59 goat samples were obtained from the Kasur district, 55 from the Okara district, and 44 from the Pakpattan district. For sheep, 41 samples were obtained from the Kasur district, 45 from the Okara district and 56 from the Pakpattan district. Informed consent was obtained from all the animal owners. The samples were taken from the vein (jugular) with sterilized syringes and shifted into a gel clot tube. Each sample was centrifuged at 3,500 rpm for 4 min and serum was collected in an Eppendorf tube and stored at -4 ^◦^C. *C. burnetii* specific antibodies were detected in sera samples using a commercially accessible indirect ELISA (IDEXX Laboratories, Switzerland) for the interpretation of results according to the instructions of the manufacturer. The ELISA employs *C. burnetii* Nine Mile as antigen and detects IgG PhI and PhII antibodies simultaneously.

### Geographic information system (GIS) mapping

Spatial dispersal of Q fever of 60 villages of 9 tehsils and 3 districts was evaluated by Arc GIS 10.5.1 software designed by Michael Scholl Meyer, Seattle, WA, USA. The facility for GIS mapping and spatial analysis was provided by the GIS laboratory of the university.

### Statistical analysis

On the basis of the Chi-square test Q fever seroprevalence and the significance of the relationship among seropositive cases and risk factors were determined. With the help of multiple logistic regression analysis, the 95% confidence intervals (CI), odd ratios (OD), and the link between factors and seroprevalence of Q fever were determined. The *p*-value was considered statistically significant when ≤ 0.05. SPSS software version 21.0 was used for particular analyses.

## Results

Three hundred samples were collected from the study area and 34 were positive for *C. burnetii* antibodies. The district Kasur showed the highest seroprevalence (14%) in sheep and goats when compared to other districts. While on the basis of tehsil, the highest seroprevalence (24%) was detected in tehsil Chunian. No seropositive sample was detected from Kot Radha Kishan (Table 1).

**Table 1:** Tehsil-wise prevalence of antibodies of *C. Burnetii* in small ruminants

Parameter	Variables	Samples Examined	Seroprevalence (%)	Chi-Square Value	***P***-Value
Tehsil	Kasur	25	4(16)	9.868	0.274
	Chunian	25	6(24)		
	Kot Radha Kishan	25	0(0)		
	Pattoki	25	4(16)		
	Okara	40	3(7.5)		
	Depalpur	30	3(10)		
	Renala khurd	30	3(10)		
	Arifwala	50	4(8)		
	Pakpattan	50	7(14)		

**Fig. 1:**
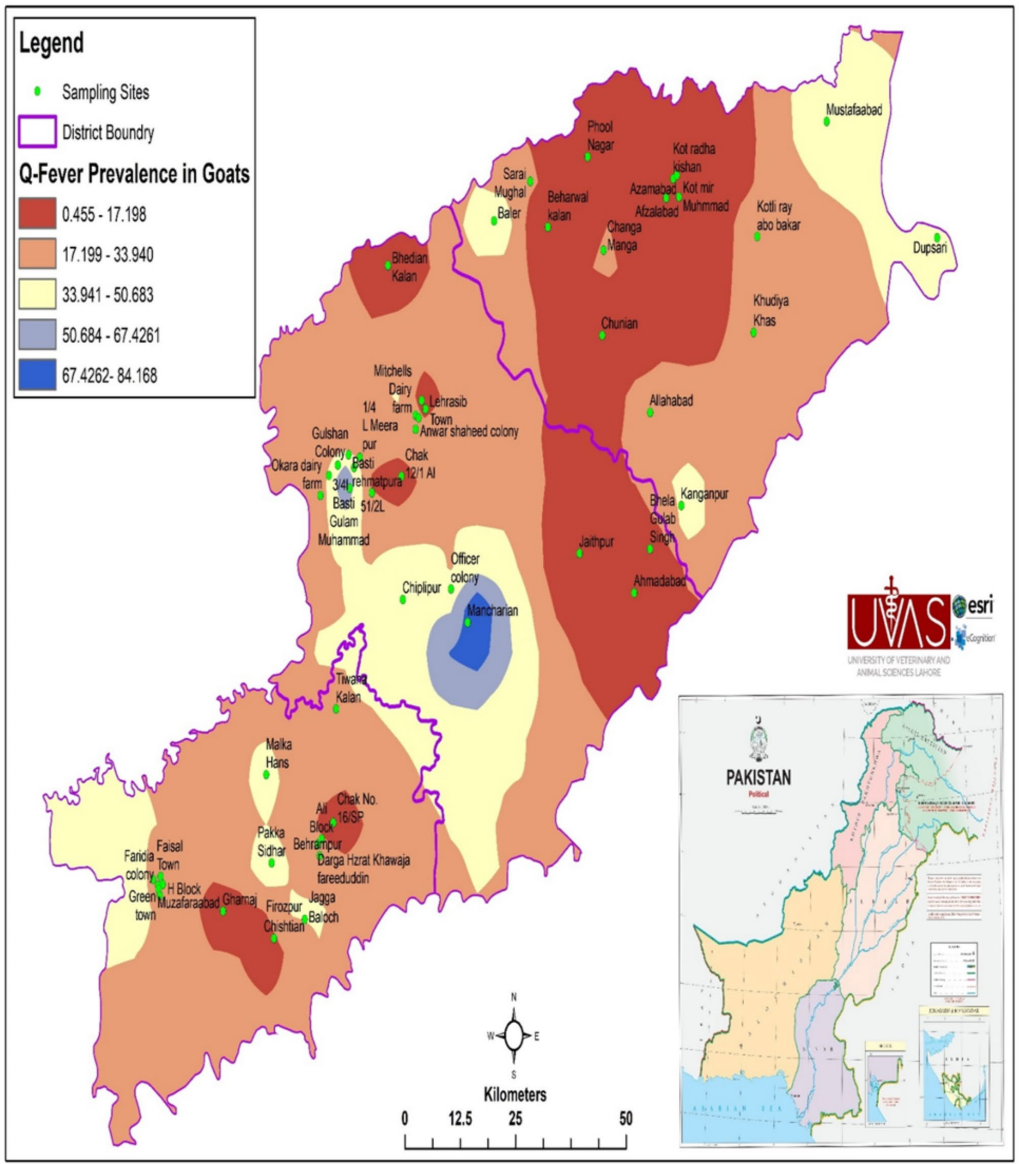
Spatial distribution and trends of Q fever in goats in selective villages of three districts of Punjab Pakistan

There was no effect of tehsil on seropositivity for antibodies of *C. burnetii*. The spatial seroprevalence and dispersal of Q fever in goats are given in Table 2. Out of 53 villages, 24 (45.3%) were found seropositive for goat samples. From tehsil Pattoki, the village Baler (100%) had the highest seroprevalences in goats. Basti Ghulam Muhammad village (100%) of the Okara tehsil, the Mancharian region (100%) from Depalpur tehsil, and the village of Mitchells dairy farm (100%) had the highest seroprevalences of their tehsils, respectively. All five villages from Kot Radha Kishan tehsil were negative. Green town, Faridia colony, Muzaffarabad, and Green town from tehsil Arifwala displayed 25%, 33.3%, and 50% respectively. 50% of goat sera from the villages Jagga Baloch and Darga Hazrat Khawaja Fareed Uddin of tehsil Pakpattan contained anti- *C. burnetii* antibodies (Table 2; Fig. 1).

**Table 2:** The influence of host species (goat) and village on the seropositivity for *C. Burnetii*

District	Tehsil	Village Name	Examined	Seropositive	Prevalence %
Kasur	Kasur	Dupsari	2	1	50
		Bhedian Kalan	1	0	0
		Mustafa Abad	2	1	50
		Kotli Ray Abo Bakar	4	1	25
		Khudian Khas	5	1	20
	Chunian	Allahabad	4	1	25
		Kanganpur	2	0	0
		Chunian	5	0	0
		Changa Manga	4	1	25
	Kot Radha Kishan	Kot Radha Kishan	3	0	0
		Kot Mir Muhammad	5	0	0
		Azamabad	2	0	0
		Afzalabad	4	0	0
	Pattoki	Phool Nagar	5	0	0
		Beharwal Kalan	3	0	0
		Sarai Mughal	5	1	20
		Baler	3	3	100
Okara	Okara	Rehman colony	1	0	0
		Gulshan Colony	4	1	25
		¾l	1	0	0
		Basti Rehmatpura	3	0	0
		1/4L Meera pur	4	0	0
		51/2L	4	0	0
		Basti Gulam Muhammad	1	1	100
		Okara dairy farm	4	1	25
	Depalpur	Officer colony	4	1	25
		Ahmadabad	3	0	0
		Bhela Gulab Singh	3	0	0
		Chiplipur	2	1	50
		Jaithpur	3	0	0
		Mancharian	1	1	100
	Renala Khurd	Chak 21/2l	2	0	0
		Mitchells Dairy farm	1	1	100
		Chak 51 RA	3	0	0
		Chak 12/1 Al	5	0	0
		Lehrasib Town	4	0	0
		Anwar Shaheed colony	2	1	50
Pakpattan	Arifwala	Faisal Town	3	0	0
		H Block	3	0	0
		Faridia colony	3	1	33.3
		Muzaffarabad	2	1	50
		Green town	4	1	25
		Gulshan Rafique Colony	1	0	0
	Pakpattan	Firozpur Chishtian	1	0	0
		Gharnaj	3	0	0
		Behrampur	5	1	20
		Jagga Baloch	2	1	50
		Pakka Sidhar	2	0	0
		Chak No. 16/SP	3	0	0
		Darga Hzrat Khawaja Fareed Uddin	2	1	50
		Ali Block	2	0	0
		Tiwana Kalan	3	1	33.3
		Malka Hans	5	2	40

**Fig. 2:**
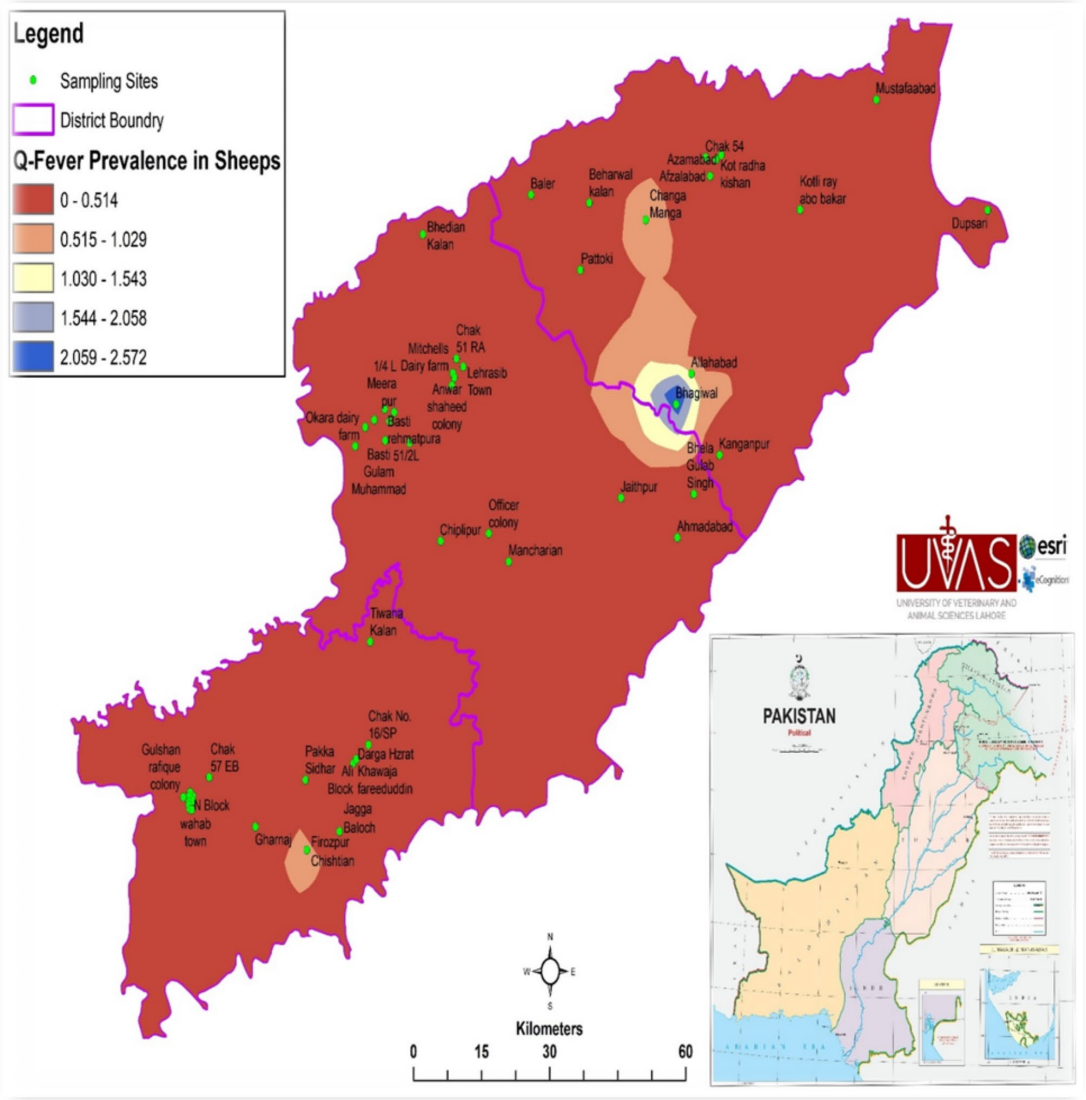
Spatial trends of Q fever in sheep in selective villages of selective districts of Punjab Pakistan

The Q fever seroprevalence in sheep is given in Table 3. Out of 52 villages, 5 (9.6%) were found to house seropositive sheep. The highest seroprevalences were observed in Bagiwal (60%) and Changa Manga (100%) villages from Chunian tehsil, respectively. No seroprevalence sheep were found in the villages of tehsils Kasur, Pattoki, Kot Radha Kishan, Okara, and Depalpur (Table 3). Although many samples were tested from tehsil Renala Khurd, Okara district, only the village Anwar Shaheed colony (33.3%) housed seropositive sheep. Only sheep of the two villages i.e. Chak 57 EB (20%) from tehsil Arifwala and Firozpur Chishtian (25%) from tehsil Pakpattan proved seropositive (Table 3; Fig. 2).

**Table 3:** The influence of species (sheep) and villages on the seropositivity for *C. Burnetii*

District	Tehsil	Village Name	Examined	Seropositive	Prevalence %
Kasur	Kasur	Dupsari	3	0	0
		Bhedian Kalan	4	0	0
		Mustafa Abad	3	0	0
		Kotli Ray Abo Bakar	1	0	0
	Chunian	Allahabad	1	0	0
		Kanganpur	3	0	0
		Bagiwal	5	3	60
		Changa Manga	1	1	100
	Kot Radha Kishan	Chak 54	5	0	0
		Kot Radha Kishan	2	0	0
		Azamabad	3	0	0
		Afzalabad	1	0	0
	Pattoki	Pattoki	5	0	0
		Beharwal Kalan	2	0	0
		Baler	2	0	0
Okara	Okara	Rehman colony	4	0	0
		Gulshan Colony	1	0	0
		¾l	4	0	0
		Basti Rehmatpura	2	0	0
		¼ L Meera pur	1	0	0
		51/2L	1	0	0
		Basti Gulam Muhammad	4	0	0
		Okara dairy farm	1	0	0
	Depalpur	Officer colony	1	0	0
		Ahmadabad	2	0	0
		Bhela Gulab Singh	2	0	0
		Chiplipur	3	0	0
		Jaithpur	2	0	0
		Mancharian	4	0	0
	Renala Khurd	Chak 21/2l	3	0	0
		Mitchells Dairy farm	4	0	0
		Chak 51 RA	2	0	0
		Lehrasib Town	1	0	0
		Anwar Shaheed colony	3	1	33.3
Pakpattan	Arifwala	M Block	5	0	0
		Faisal Town	2	0	0
		N Block	5	0	0
		H Block	2	0	0
		Faridia colony	2	0	0
		Muzaffarabad	3	0	0
		Green town	1	0	0
		Gulshan rafique colony	4	0	0
		Chak 57 EB	5	1	20
		Wahab town	5	0	0
	Pakpattan	Firozpur Chishtian	4	1	25
		Gharnaj	2	0	0
		Jagga Baloch	3	0	0
		Pakka Sidhar	3	0	0
		Chak No. 16/SP	2	0	0
		Darga Hazrat Khawaja Fareed Uddin	3	0	0
		Ali Block	3	0	0
		Tiwana Kalan	2	0	0

For the analysis of the geographical distribution of Q fever, various factors were investigated. No male sheep was seropositive but 19.2% of the male goats had anti- *C. burnetii* antibodies. The risk to attain the pathogen was greater in female goats as compared to female sheep (Table 4). The spatial distribution and trends of the Q fever seroprevalence of the sheep populations of various villages of Punjab are shown in Fig. 2. No sample of sheep younger than one year of age. Four (8.51%) of the 47 sheep of the 1–2 years age group were seropositive while seroprevalences of 6.9%, and 1.8% were found in the 2–3 years and above three years group, respectively (Table 5). Based on multiple logistic regression analysis, ‘gender’ was considered as a potential risk factor in sheep. In goats ‘age’ was considered a significant risk factor (Table 6). In contrast to the sheep population, the highest reported seroprevalence was found in the three-year age group.

**Table 4:** The influence of species and gender on the seropositivity for *C. Burnetii*

Species	Gender	Examined	Seropositive	Prevalence%	Chi-square	***P*** value
Sheep	Male	14	0	0	0.805	0.476
	Female	128	7	5.5		
Goat	Male	26	5	19.2	0.101	0.47
	Female	132	22	16.7		

**Table 5:** The impact of the age factor and type of animals for the Q fever prevalence in three districts of Punjab Pakistan

Species	Age (years)	Examined	Seropositive	Prevalence (%)	Chi-square	***P*** value
Sheep	< 1	11	0	0	0.232	0.357
	1–2	47	4	8.51		
	2–3	29	2	6.9		
	>3	55	1	1.8		
Goat	< 1	18	0	0	8.574	0.036
	1–2	61	9	14.8		
	2–3	20	7	35		
	>3	59	11	18.6		

**Table 6:** Association of gender and age of an animal with Q fever using multiple logistic-regression analysis

Species	Variable	Factors	***P***- value	Odd Ratio	95% CI	
					Lower Bound	Upper Bound
Sheep	Gender	Female	0.0	25868619.9		25868619.92
		Male		Ref.		
	Age	< 1year	0.28	3.852	0.334	44.417
		1–2 year	0.998	2.04E-07		0
		2–3 year	0.118	5.943	0.637	55.423
		> 3		Ref.		
Goat	Gender	Female	0.464	0.656	0.212	2.028
		Male		Ref.		
	Age	< 1 year	0.057	1.29E-09	1.29E-09	1.29E-09
		1–2 year	0.54	0.739	0.281	1.946
		2–3 year	0.13	2.396	0.772	7.437
		> 3		Ref.		

## Discussion

Q fever is a zoonotic disease with major reservoirs in domestic ruminants and is of importance for public veterinary services and public health thus. Production losses i.e. abortions, stillbirth and reduced milk yield of livestock negatively affect the prosperity of farmers. Animals may shed the causative agent, *Coxiella burnetii*, in high numbers with milk and birth products putting other animals and humans at high risk of infection and contaminate surrounding environments. The here employed ELISA detects long-lasting IgG antibodies specific for *C. burnetii*. It detects both antigenic forms, termed phase I and phase II, simultaneously, but reported diagnostic sensitivities (DSe) and diagnostic specificities (DSp) vary widely [32, 33]. However, the test has a high specificity above 99% and therefore a low false positive error [34]. This test kit is licensed in Germany for veterinary diagnostic purpose and represents a suitable test for prevalence estimation. This spatial study investigated the connections between Q fever and small ruminants in various villages in Punjab Pakistan. There were striking differences in the seroprevalence of sheep and goats between villages noted i.e. 24% in Chunian tehsil vs. 0% in Kot Radha Kishan. It can be assumed that contact of these animals with other infected animals was limited as they remained bounded to the respective villages of this tehsil. The current study also found a higher prevalence in goats than in sheep. Goats may function as epizootic hosts in the local setting of Punjab as can be supposed by epidemiologic data from other countries. Goats have a 3- and 2.27-times chance of getting infected than sheep or cattle, respectively. Thus, goats are more vulnerable than other farm animals. During a massive outbreak of Q fever in the Netherlands, Q fever spread was linked to clinically diseased goats [9]. High seroprevalences in goats were also found in southeast Ethiopia 54.2% [35, 36], in Iran 33% [16], in USA 41.6% [37] and in Saudi Arabia 34.04% [38], 15.6% [39]. In all these countries the seroprevalence in sheep was noticeable variable. Sheep have to be considered as reservoir as well in many countries and should not be neglected during control programs in small ruminants as shown by data from Turkey 20% [40], Egypt 33.2% [41], Paraguay 45% [42], Greece 10.4% [43] and Great Britain 0.9% [44]. Hence, different studies can only be compared with caution as wide fluctuations for Bulgaria, France and Germany i.e. 56.9%, 20% and 8.7% respectively, are reported [45]. Prevalence studies are done often after outbreaks and thus higher values may not be unexpected. Thus, it is not surprising that higher values may be reported for sheep when compared to that of goats e.g. in Greece 10.4% vs. 6.5% [46], in Egypt 22.5% vs. 16.8% [47]. Small ruminants are always to be considered as sources for Q fever outbreaks e.g. in Bosnia and Herzegovina multiple outbreaks were traced back to goats and/or sheep [46, 48]. Local climate is a factor to be considered in Q fever epidemiology as pathogens are highly resistant to harsh climatic conditions. Dry season urges goats to roam more freely in vast areas and thus these animals come more often in contact with infected ruminants from other villages spreading the disease. Sheep usually remain closer to the villages and they stay in limited areas. This can be one of the reasons why sheep may be less prone to Q fever than goats [49]. Climate and animal density were identified to be risk factors for outbreaks of this pathogen and seroprevalence in earlier studies [41, 50].

The seroprevalence of Q fever was higher in male goats (19.2%) as compared to females (16.7%). In a recent Pakistani study a higher prevalence was observed in females but this finding was not significant [23]. In a study from China, a seroprevalence of 4.68% was reported in male goats and 4.81% in female goats [17]. Both sexes obviously seem to be at the same risk for infection but this finding further research involving statistically relevant numbers of animals of both sexes. Various authors have recorded data corroborating the finding that adult animals are more often (effectively) exposed to Q fever than younger ones and thus are more often found to harbor anti-*C. burnetii* antibodies [38, 40, 51, 52]. These later studies also show that 1–2 years old animals have a direct impact on infection due to increased abortion rates. Vertical transmission may be another reason for fewer cases in young animals [52]. In Pakistan humans and animals are vulnerable to Q fever. Thus, Q fever indeed will cause considerable damage to many socioeconomic aspects of animal farming and meat industry. Moreover, the threat to human life cannot be neglected as the whole industry still relies on extensive farming practices which require intensive contact between the animal and its human counterpart.

## Conclusions

*Coxiella burnetii*, the causative agent of zoonotic Q fever is present in sheep and goats of the Pakistani Punjab and poses a stead risk of transmission to humans and other animals. The spatial analysis highlights the ubiquitous distribution of Q fever in all study areas. Preventive measures should be taken while handling the animals and their products.

## Data Availability

Data is provided within the manuscript.
